# Why Is Diabetes Mellitus a Risk Factor for Contrast-Induced Nephropathy?

**DOI:** 10.1155/2013/123589

**Published:** 2013-11-21

**Authors:** Samuel N. Heyman, Christian Rosenberger, Seymour Rosen, Mogher Khamaisi

**Affiliations:** ^1^Department of Medicine, Hadassah Hospital, Mt. Scopus and the Hebrew University Medical School, P.O. Box 24035, Jerusalem 91240, Israel; ^2^Department of Nephrology, Charité Campus Mitte, Berlin 10115, Germany; ^3^Department of Pathology, Beth Israel Deaconess Medical Center and Harvard Medical School, Boston, MA 02215, USA; ^4^Joslin Diabetes Center, Harvard Medical School, Boston, MA 02215, USA

## Abstract

Contrast-induced nephropathy (CIN) remains a leading cause of iatrogenic acute kidney injury, as the usage of contrast media for imaging and intravascular intervention keeps expanding. Diabetes is an important predisposing factor for CIN, particularly in patients with renal functional impairment. Renal hypoxia, combined with the generation of reactive oxygen species, plays a central role in the pathogenesis of CIN, and the diabetic kidney is particularly susceptible to intensified hypoxic and oxidative stress following the administration of contrast media. The pathophysiology of this vulnerability is complex and involves various mechanisms, including a priori enhanced tubular transport activity, oxygen consumption, and the generation of reactive oxygen species. The regulation of vascular tone and peritubular blood flow may also be altered, particularly due to defective nitrovasodilation, enhanced endothelin production, and a particular hyperresponsiveness to adenosine-related vasoconstriction. In addition, micro- and macrovascular diseases and chronic tubulointerstitial changes further compromise regional oxygen delivery, and renal antioxidant capacity might be hampered. A better understanding of these mechanisms and their control in the diabetic patient may initiate novel strategies in the prevention of contrast nephropathy in these susceptible patients.

## 1. Introduction

**Figure 1:**
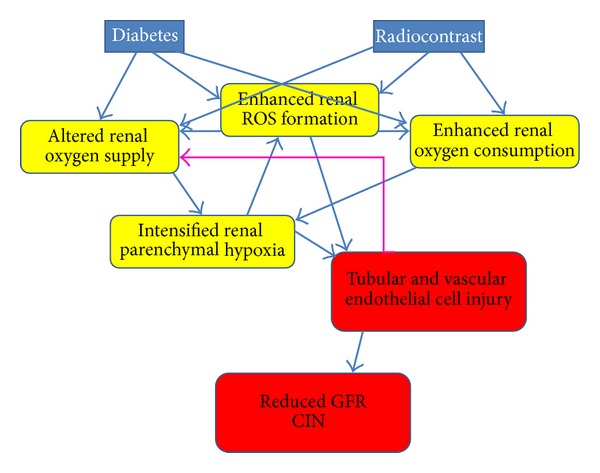
Plausible synergic adverse impact of radiocontrast agents and diabetes upon the kidney, leading to contrast-induced nephropathy (CIN). Both conditions, diabetes and the administration of iodinated radiocontrast agents, lead to altered renal physiologic processes (in yellow): there is an excess formation of reactive oxygen species (ROS) and altered renal oxygenation, related to disregulated renal microcirculation and enhanced tubular transport and oxygen consumption. Evolving renal parenchymal hypoxia and enhanced ROS formation lead to tubular and vascular endothelial injury, with subsequent reduction of glomerular filtration rate (GFR), the hallmark of CIN. Conceivable interactions between these processes are outlined by arrows and discussed in depth in the text. In brief, both diabetes and contrast agents enhance ROS formation. They also hamper renal oxygenation, either directly or through increased generation of ROS. Vascular endothelial cell injury may further amplify renal hypoxia via a feed-forward loop of altered microcirculation (green arrow).

Contrast-induced nephropathy (CIN) induced by iodinated contrast media remains a leading cause of inhospital acute kidney injury (AKI), despite the introduction of new low- or iso-osmolar agents and the implementation of preventive measures. This reflects the growing usage of computerized imaging and intravascular interventions in sicker and older patients [1]. The pathophysiology of CIN is complex and only partially understood. While the role of a direct nephrotoxicity affecting tubular cells is debatable, substantial evidence indicates that renal parenchymal hypoxia, particularly within the renal outer medulla, and the generation of reactive oxygen species (ROS) play a pivotal role in this disorder, as reviewed in depth elsewhere [1–3] and illustrated in Figure 1.

While healthy individuals seldom develop CIN, the well-defined risk factors are associated with increasing likelihood to acquire this complication. The likelihood of CIN rises with the number of predisposing factors, and algorithms were designed to predict the risk of CIN, based on the presence of these defined predisposing factors [4]. Chronic kidney disease (CKD) with impaired glomerular filtration rate (GFR) is the most important intrinsic predisposing factor. The risk to develop CIN is proportional to the degree of renal impairment, and in patients with a GFR of about 10–15 mL/min, it may exceed 50%. Diabetes is an additional important risk factor, and for every given baseline GFR in patients with CKD, the presence of diabetes was found to double the risk to develop CIN [5]. Multivariate analysis of database of 8357 patients defined diabetes as an independent risk factor with an odd ratio of 1.6 [4], and prediabetes was found to increase the risk of CIN [6]. Since both renal impairment and diabetes are prevalent among patients subjected to radiocontrast media, a better understanding of the nature of this disorder, particularly in their presence, is important and is the objective of this review.

## 2. Renal Hypoxia and Oxidative Stress and Their Role in CIN

The special structure of the renal medulla, designed for urinary concentrating capability, is the cause for low outer medullary oxygen tension (pO_2_), at the range of 30–40 mmHg under normal physiologic conditions. Low medullary oxygenation reflects intense tubular transport (particularly by medullary thick limbs, mTALs) in a region with limited blood and oxygen supply [7]. Highly efficient neurohumoral mechanisms maintain barely balanced medullary oxygenation by matching regional tubular transport activity and blood supply. Medullary tubular transport activity is governed by solute delivery to the distal nephron (determined by GFR and proximal tubular transport) and by the direct regulation of mTAL transporters, particularly Na-K ATPase. Blood and oxygen supply, delivered through peritubular capillaries, depends on limited blood flow through vasa recta that emerge from juxta-medullary nephrons. Adenosine, nitric oxide (NO), prostaglandins (particularly PGE_2_ and PGI_2_), and other agents maintain medullary oxygenation by reducing transport activity, by enhancing regional blood flow, or by a combination of both [7, 8].

As reviewed in depth [1, 2], the intravascular administration of all types of iodinated radiocontrast agents further reduces medullary pO_2_ to levels as low as 15–20 mmHg. This has been clearly documented under experimental settings with oxygen microelectrodes, by the detection of pimonidazole adducts (which bind to tissues with pO_2_ below 10 mmHg) or by the regional accumulation of hypoxia-inducible factors (HIFs). HIFs are key regulators of cellular response to hypoxic stress, controlling the expression of numerous genes involved in cell metabolism, proliferation, and survival and in the structure and function of regional microcirculation. Under physiologic conditions, HIF subunit *α* undergoes proteasomal degradation, initiated by oxygen-sensitive HIF-prolyl hydroxylases. Intensified hypoxia de-activates HIF-prolyl hydroxylases, leading to HIF-*α* accumulation and its binding to HIF-*β* subunits. The formed HIF-*αβ* heterodimer undergoes nuclear translocation, binds to hypoxia-response elements (HRE) along the DNA strands, and initiates transcriptional responses [9]. Indeed, nuclear HIF expression has been detected in medullary structures following the administration of radiocontrast media [10], indicating the induction of cellular hypoxic stress response by contrast agents.

Importantly, this is a sublethal form of hypoxic stress [11], since it does not lead to structural damage or to renal dysfunction in intact animals. This resembles healthy individuals that hardly ever develop CIN. By contrast, CIN may develop when animals are preconditioned by measures that mimic clinical conditions predisposing to CIN [2, 12]. Since many of these comorbidities are characterized by altered regulatory systems that maintain medullary regional oxygenation during hypoxic stress, simplified animal models were developed such as the inactivation of cyclooxygenase and NO synthase (NOS) prior to radiocontrast injection [13]. In these models, inhibition of prostaglandin or NO synthesis abolishes outer medullary vasodilatory response to the radiocontrast and intensifies medullary hypoxia, leading to AKI with outer medullary hypoxic damage. Tubular injury, ranging from apoptosis to frank necrosis, mostly affects mTALs in the inner stripe of the outer medulla. However, in the most severe models damage may extend outwards to the outer stripe and medullary rays and inwards into the papilla, involving S3 segments of the proximal tubules, as well as thin limbs, and collecting ducts, respectively. Within the outer medulla, a peculiar gradient of hypoxic injury is noted, increasing from the better oxygenated region around vasa recta to the mid-interbundle zone, most remote from oxygen supply [1, 12].

The complex mechanisms involved in the decline in medullary oxygenation following the administration of contrast media are discussed in depth elsewhere [1, 2]. In brief, on one hand, oxygen consumption for tubular transport may increase due to a transient surge in GFR and the diuresis following radiocontrast administration. Furthermore, enhanced ROS formation increases mTAL transport activity and oxygen expenditure (see below). On the other hand, medullary regional microcirculation and oxygen delivery decline, as the result of increased interstitial pressure with capillary collapse and due to altered regulation of vascular tone. This later phenomenon is believed to be principally related to an increase in adenosine generation and endothelin synthesis and to a decline in NO availability [1, 2]. Finally, direct endothelial cell injury, with extravasation of red blood cells, has recently been noted in the inner stripe of the outer medulla of mice subjected to contrast agents [14] complementing previous findings of radiocontrast-induced endothelial cell damage *in vitro* [15]. The only preconditioning in these animals was dehydration. These findings raise the possibility that hypoxic or toxic damage to vascular endothelial cells may also play a pivotal role in the evolution of medullary hypoxia under certain settings.

As reviewed elsewhere in depth [1, 3], the administration of contrast media results in enhanced formation of ROS and with oxidative and nitrosative stress that likely participate in the pathogenesis of CIN. Conceivably, it is the combination of enhanced transport activity and hypoxia that generates ROS, as most *in vitro* studies with renal cells or isolated nephron segments failed to demonstrate a direct impact of radiocontrast upon ROS generation. ROS alter renal medullary oxygen balance by both the enhancement of tubular transport and oxygen consumption and by disrupting nitrovasodilation. The importance of ROS in the alteration of renal microcirculation is underscored by the *in vitro* amelioration of radiocontrast-induced vasa recta constriction in response to antioxidants [16].

## 3. Evidence for Intensified Hypoxia and Oxidative Stress in the Diabetic Kidney

The predisposition of the diabetic kidney to CIN might be related to intensified renal hypoxia and oxidative stress, as reviewed in depth in [17]. Indeed, experimental diabetes is associated with reduced renal pO_2_. Using oxygen microelectrodes, Palm and colleagues [18] demonstrated lower renal parenchymal pO_2_ in diabetic rats, as compared with control animals, both in the cortex (about 36 versus 50 mmHg) and in the medulla (around 11 versus 27 mmHg). This observation was also confirmed noninvasively with BOLD MRI, detecting increasing concentrations of deoxygenated hemoglobin in the outer medulla in diabetic animals [19, 20]. Furthermore, pimonidazole adducts (which indicate a pO_2_ <10 mmHg) were detected in the renal outer medulla and papilla in rats within 7 days after the induction of diabetes with streptozotocin, while untreated controls had no evidence of pimonidazole accumulation. Evolving medullary hypoxia was associated with HIF stabilization in these regions, indicating tissue reaction to hypoxic stress [21]. Chronic HIF stimulation in the diabetic kidney may also be triggered by regional inflammation [22, 23].

Diabetes is associated with enhanced ROS formation, which is believed to play an important role in diabetic nephropathy. As discussed in depth elsewhere [17], enhanced ROS in the diabetic kidney is principally due to the increased activity of NADPH oxidase and the augmented mitochondrial generation of superoxide, related to increased metabolic activity and the glycation of mitochondrial proteins. Activation of the renin-angiotensin axis may also intensify NADPH oxidase in the diabetic kidney [17].

## 4. Mechanisms of Augmented Hypoxia in the Diabetic Kidney: Enhanced Oxygen Consumption

As thoroughly reviewed elsewhere [24], enhanced oxygen consumption for tubular solute reabsorption is a major determinant in the development of hypoxia in the diabetic kidney. Early diabetes is associated with a marked increase in GFR, in parallel with kidney growth and increased glomerular and tubular mass, either by hyperplasia or hypertrophy [25], implying greater oxygen utilization. Increased solute delivery to the nephron, augmented tubular transport, and oxygen consumption reflects enhanced GFR and osmotic diuresis [1, 2]. Indeed, in analogy, experimental renal hypertrophy, induced by uninephrectomy or with high protein diet, enhances renal medullary Na-K-ATPase content and predisposes to outer medullary hypoxic injury in isolated rat kidneys, perfused with red-cell-free oxygenated medium [26]. Prior uninephrectomy with hypertrophy of the remnant kidney was also found to be an important independent contributor to the development of AKI *in vivo* in rats subjected to salt depletion, indomethacin, and radiocontrast [27]. An injury pattern was noted, comparable to that in the isolated perfused kidney, principally with mTAL necrosis in the outer medulla. In the same manner, acutely enhancing GFR with the infusion of glycine resulted in augmentation of outer medullary injury and renal dysfunction in the same experimental model of CIN [28].

It is tempting to assume that in advanced diabetic nephropathy with reduced numbers of nephrons, oxygen consumption for tubular transport per remnant nephron might be larger, with a greater risk for the development of critical hypoxia. Hyperglycemia and diabetes increase Na/glucose-linked transporters; enhance expression of uncoupling protein-2; and augment free fatty acid metabolism, gluconeogenesis, and glycogen synthesis, hence increasing oxygen consumption by tubular cells [17, 22]. Furthermore, increased ROS generation in the diabetic kidney enhances Na-K-2Cl cotransport activity, as shown in isolated mTALs [29], while impeding electrolyte transport efficacy (i.e., more oxygen demand per transport load) [22, 24]. Indeed, Palm et al., exploring the diabetic kidney, found that the addition of antioxidants reduced tubular oxygen consumption *in vitro* and attenuated renal parenchymal hypoxia *in vivo* [18]. Thus, in the diabetic kidney a feed-forward loop likely exists, of regional hypoxia, enhanced ROS generation, and increased tubular transport, which further amplifies regional hypoxia. The administration of contrast media may step up this devastating process in the diabetic kidney by further intensification of hypoxia and ROS formation.

## 5. Mechanisms of Augmented Hypoxia in the Diabetic Kidney: Altered Oxygen Supply

*Macrovascular* disease and chronic tubulointerstitial changes likely compromise oxygen delivery and diffusion in advanced diabetes and diabetic nephropathy, hence predisposing to CIN. In addition, it is tempting to assume that diabetic autonomic neuropathy disrupts the capacity to preserve renal perfusion pressure and alters renal innervation involved in the maintenance of intrarenal distribution of blood flow and oxygen supply. An additional plausible mechanism for intensified hypoxia in the diabetic kidney is an increase in oxygen shunt from descending to ascending vasa recta, as a result of the Bohr effect, related to reduced interstitial pH [30].

Furthermore, the regulation of various mediators which determine renal vascular tone and microcirculation are hampered in the diabetic kidney, leading to a greater susceptibility to the development of critical hypoxia following the administration of contrast material. Principal factors likely involved are faulted nitrovasodilation, enhanced endothelin synthesis, and increased effect of adenosine.

### 5.1. Altered NO Generation

The complex interplay between NO, ROS, and oxygen supply and utilization [17, 31] is beyond the scope of this review. Yet, importantly, as noted above, the diabetic kidney is characterized by enhanced ROS generation, altered NO response, and reduced pO_2_. Renal medullary NO is low in the diabetic kidney [18, 32]. Conceivably, increased ROS generation reduces NO availability in these settings through its scavenging, with the formation of the highly toxic peroxynitrite. Additional contributors to NO depletion in the diabetic kidney are NOS uncoupling, reduced membranal eNOS activity, and increased levels of PKC, ADMA, and intracellular arginase with consequent L-arginine depletion [17, 32].

Renal NO abruptly rises in the normal rat kidney following radiocontrast injection [33], and its significance in maintaining renal oxygenation in these settings is underscored by the conversion of medullary vasodilatory response into vasoconstriction following the administration of radiocontrast, at the presence of NOS inhibition [13]. Palm et al. noted that the administration of antioxidants not only reduced oxygen consumption for tubular transport in the diabetic kidney, but also somewhat improved outer medullary microcirculation [18]. Thus, ROS-mediated NO depletion might be harmful to the renal microcirculation following radiocontrast administration, especially in the diabetic kidney.

### 5.2. Increased Endothelin Synthesis

The administration of contrast media is associated with increased plasma levels of endothelin-1 (ET-1) under experimental settings [34]. ET-1 levels also increase following radiocontrast administration and are higher both at baseline and following radiocontrast in diabetics and in patients with CKD [35]. Diabetes is associated with enhanced synthesis of the pre-pro-hormone. In addition, endothelin-converting enzyme (ECE)-1, which catalyzes the conversion of the prohormone to the active compound ET-1, is also upregulated in diabetes. Experimental diabetes in rats resulted in a fivefold increase in ECE-1 expression in the outer medulla, and a 15-fold increment was noted in diabetic rats subjected to contrast medium [36]. Conceivably, radiocontrast-induced enhanced production of ET-1, particularly in the diabetic kidney, by both the induction of prehormone and ECE-1 synthesis, participates in microvascular dysregulation and in the development of CIN. PKC, activated in diabetes [37], is a known mediator of ECE-1 activation [38]. Furthermore, ECE-1 is likely a HIF-target gene [39], and possibly enhanced medullary ECE-1 is triggered by HIF, induced by both diabetes and contrast media.

### 5.3. Altered Generation and Action of Adenosine

As outlined thoroughly elsewhere [40], adenosine has a marked influence upon renal parenchymal oxygenation, playing a central role in the tubuloglomerular feedback mechanism, affecting both tubular transport activity and the renal microcirculation. The effects of adenosine are highly complex and depend on the distribution and action of its receptors, on adenosine concentrations, and on the duration of receptor stimulation. Adenosine-induced enhancement of proximal tubular reabsorption and inhibition of mTAL transport activity improve medullary oxygenation. However, its microvascular effects are more complex regarding medullary oxygenation, exerting both vascular constriction (via adenosine A1 receptors) and vascular dilatation (stimulated by adenosine A2 receptors). Some vascular beds, such as descending vasa recta, express both types of receptors [40]. The diverse and complex effects of adenosine on the renal microcirculation can be demonstrated by its exogenous administration in the presence of selective adenosine inhibitors, illustrating an overall reduction of cortical microcirculation and oxygenation and amelioration of medullary hypoxia [41]. Yet endogenously generated adenosine may exert quite different vascular responses, and altered NO synthesis or increased angiotensin II might modify their overall activity, potentiating vasoconstriction [40].

Adenosine, generated following radiocontrast administration by ATP breakdown in the hypoxic kidney, conceivably participates in the pathogenesis of CIN. Indeed, adenosine nonselective or A1 receptor-selective blockers or knock-out manipulations were found to blunt the decline in renal blood flow and GFR and to attenuate CIN in experimental models and in humans [42–52].

Endogenous generation of adenosine might be modified in the hypoxic diabetic kidney, and its effects upon renal microcirculation and oxygenation may be altered, particularly in the presence of altered NO and enhanced AII synthesis. Indeed, exogenous adenosine was found to markedly intensify renal vasoconstriction in diabetic rats as compared with intact animals [53]. Furthermore, as compared to intact controls, diabetic animals displayed attenuation of vasoconstrictive response to adenosine following the inhibition of prostaglandin [54] or NO synthesis [55], suggesting a diminished vasodilatory capacity of NO or prostaglandins (PGE_2_, PGI_2_) to counteract adenosine-induced vasoconstriction.

## 6. Contrast Nephropathy in the Diabetic Kidney: Controversies and Pitfalls

As outlined above, the propensity to develop CIN in the diabetic kidney might be related to both lower baseline medullary pO_2_ and enhanced ROS formation, with altered tubular cellular metabolism and microcirculation. It is anticipated that radiocontrast administration, also characterized by hypoxic and oxidative stress, by its own should further amplify renal hypoxic and oxidative stress in the diabetic kidney. Yet, few observations do not fit well with this concept, underscoring the complexity of these settings.

There are many confounders both in clinical and experimental settings that may blur our understanding, such as the type and duration of diabetes, and the degree of renal impairment and microvascular compromise. In experimental settings, interspecies differences and the model types of diabetes and CIN might play a role. In both experimental and clinical studies, there is an impact of the type of contrast media, their volume and mode of administration, additional comorbidities such as renal failure at various stages, the administration of preventive measures, and other factors involved that are yet to be defined.

As an example for an impact of progression of diabetes along time, in streptozotocin-induced diabetes in rats, renal cortical blood flow and GFR (and conceivably oxygen demand for tubular transport) are initially increased, but decline by 4–9 weeks. Accordingly, intensified medullary hypoxia; enhanced pimonidazole immunostaining; and HIF expression, noted at earlier time points, tend to decline over time [21, 56]. At this later time point, radiocontrast administration, though altering medullary blood flow, did not further reduce medullary pO_2_, as opposed to a marked decline in control animals [56].

The potential confounding impact of preventive measures is illustrated in a clinical trial in patients with CKD, where renal blood flow, lower at baseline in diabetics, as compared with patients with comparable renal impairment, increased more profoundly during coronary angiography in response to renal vasodilators [57].

Additional potential confounder is chronic hypoxia adaptation in the diabetic kidney that might confer tolerance to hypoxic and oxidative stress and modify the phenotype of CIN. Unexpectedly, diabetic rats subjected to contrast medium following cyclooxygenase inhibition with indomethacin displayed only rare and focal mTAL injury in the inner stripe of the outer medulla, and the degree of kidney impairment was comparable to nondiabetic animals [58]. By contrast, diabetic kidneys perfused with cell-free oxygenated medium (intense hypoxic conditions) exhibited most severe hypoxic damage, as compared with moderate and reversible outer medullary injury in control kidneys [58]. We hypothesized that hypoxia preconditioning in the diabetic kidney, with upregulation of HIF signals and the expression of reno-protective HIF-dependent genes [21], might mitigate hypoxic damage caused by contrast media, but not under extreme hypoxic stress, as occured in the isolated perfusion system. This observation bears resemblance to the unexpected renal medullary protection against hypoxic injury in experimental CIN in animals with chronic tubulointerstitial disease, characterized by rarefaction of peritubular capillaries, medullary hypoxia and HIF induction [59]. In these settings, a more pronounced rise in plasma creatinine in CKD rats following a CIN protocol, at the absence of a morphologic counterpart, likely reflects disruption of renal hemodynamic adaptive mechanisms that at baseline compensate for the structural changes.

As the studies detailed above illustrate a potential dichotomy between changes in GFR and renal structural damage, a true assessment of the efficacy of preventive strategies in CIN in diabetic patients, outlined below, might be better understood by the determination of urine biomarkers of tubular damage [60].

## 7. Renal Hypoxic and Oxidative Stress in the Diabetic Kidney Subjected to Radiocontrast: Therapeutic Implications

Enhancement of GFR has been tried in the prevention of CIN. As reviewed above, this approach may enhance solute delivery for reabsorption along the nephron, hence more transport activity and oxygen consumption. This approach might be especially hazardous in the diabetic kidney, prone to a more profound hypoxic and oxidative stress. Indeed, in few clinical trials, enhancing GFR and diuresis was found to adversely affect kidney function in diabetic patients undergoing coronary angiography. In a small study by Weisberg et al. the administration of dopamine, atrial natriuretic factor (ANP) or mannitol to patients with CKD, resulted in a 30–50% increment in plasma creatinine in diabetics, but not in nondiabetic individuals [57]. Likewise, diabetics were particularly susceptible to develop CIN when given an ANP analogue in a randomized well-controlled study by Kurnik et al. [61]. Two-hundred and forty seven patients with CKD undergoing radiocontrast studies were enrolled, half of them were diabetics. The incidence of CIN among nondiabetics was comparable in patients given saline or saline + ANP (9% and 8%, resp.). By contrast, among diabetics, ANP treatment tended to enhance the risk of CIN (26% versus 39%, resp.). However, the potential contribution of volume depletion and prerenal azotemia to the special susceptibility of diabetics in the above studies was not unequivocally excluded. Future experiments applying strict control of euvolemia, as recently conducted in the Remedial II trial [62], are required to address this debate.

In the same fashion, the role of adenosine-receptor blockers in the prevention of CIN, based on studies showing enhanced renal blood flow and GFR warrants further consideration, since adenosine helps to maintain medullary oxygenation (though at the price of reduced GFR). Indeed, Liss and colleagues found that in normal rats medullary oxygenation fell by 24% with adenosine [63], and such an effect might be especially hazardous in the diabetic kidney.

Endothelin receptor antagonists in particular endothelin ETA receptor blockers might also be useful in the prevention of CIN [64], particularly in diabetic patients, characterized by markedly enhanced endothelin synthesis following radiocontrast administration [35, 36]. Interestingly, preliminary studies looking at the urine excretion of neutrophil gelatinase-associated lipocalin (NGAL) suggest the attenuation of distal tubular injury in diabetic patients even in the absence of exposure to radiocontrast agents [65].

Osmotic diuresis in uncontrolled diabetes theoretically might help excretion of contrast media and lower its concentration in the tubular lumen, decreasing urine viscosity and consequently reducing interstitial pressure and microvascular compromise. However, appropriate generous hydration seems to be warranted particularly in these setting to compensate for fluid deficit and to achieve sufficient volume expansion.

Since enhanced transport activity and oxygen consumption are a principal cause of renal hypoxia in the diabetic kidney, the use of loop diuretics, which ameliorates medullary hypoxia [66] and hypoxic damage in experimental contrast nephropathy [67], with careful fluid replacement to avoid volume depletion [62], might be renoprotective, particularly in diabetics, but this has yet to be evaluated.

Dyslipidemia is common among diabetic patients, and hypercholesterolemia has been identified as a predisposing factor to CIN in experimental AKI, characterized by compromised NO synthesis and enhanced ROS generation [68]. Interestingly, a loading dose of atorvastatin was recently found to attenuate the risk for CIN in CKD patients treated with NAC and bicarbonate. Yet, this effect was comparable among diabetic and nondiabetic individuals, and the mechanisms of renal protection might not involve lipid metabolism [69].

The administration of the antioxidant N-acetylcysteine and bicarbonate infusion are controversial strategies in the prevention of CIN [1–3]. The use of antioxidants in the prevention of CIN is particularly reasonable in diabetic patients, characterized by a priori enhanced ROS generation. Increasing renal parenchymal pH is also reasonable especially in the diabetic patient in order to reduce oxygen shunt across vasa recta. Antioxidants may also improve hypoxic stress response through HIF stabilization [21], which might be renoprotective. Yet, an effect of such strategies has not been explored specifically in diabetic patients, and this might be an objective in future clinical trials. Administration of L-arginine seems to be an additional plausible option in diabetic patients prior to radiocontrast studies, as its deficiency might contribute to renomedullary hypoxia [32].

## 8. Conclusions

Diabetes and the administration of iodinated radiocontrast agents are both associated with marked alterations of renal physiology, including changes in GFR and renal hemodynamics, enhanced tubular transport activity and oxygen expenditure and intensification of medullary hypoxia, and ROS generation. Diabetes conceivably predisposes to CIN principally through the amplification of these changes and the disruption of protective mechanisms, designed to maintain medullary oxygenation and to ameliorate oxidative stress. The efficacy of preventive measures in clinical trials in CKD patients should be separately assessed for this subgroup of diabetic individuals.
